# DUSP9 alleviates hepatic ischemia/reperfusion injury by restraining both mitogen-activated protein kinase and IKK in an apoptosis signal-regulating kinase 1-dependent manner: Role of DUSP9 in hepatic ischemia/reperfusion injury

**DOI:** 10.3724/abbs.2022183

**Published:** 2022-12-22

**Authors:** Zhongtang Li, Zuotian Huang, Yunhai Luo, Hang Yang, Mei Yang

**Affiliations:** 1 College of Basic Medicine Chongqing Medical University Chongqing 400016 China; 2 The First Affiliated Hospital of Chongqing Medical University Chongqing 400016; 3 Department of Hepatobiliary Pancreatic Tumor Center Chongqing University Cancer Hospital Chongqing 400030 China

**Keywords:** DUSP9, hepatic ischemia/reperfusion injury, ASK1, IKK

## Abstract

Hepatic ischemia/reperfusion (I/R) injury occurs frequently in various liver operations and diseases, but its effective treatment remains inadequate because the key switch that leads to hepatic explosive inflammation has not been well disclosed. Dual specificity phosphatase 9 (DUSP9) is widely involved in the innate immune response of solid organs and is sometimes regulated by ubiquitin. In the present study, we find that DUSP9 is reduced in mouse hepatic I/R injury. DUSP9 enrichment attenuates hepatic inflammation both *in vivo* and *in vitro* as revealed by western blot analysis and qRT-PCR. In contrast, DUSP9 depletion leads to more severe I/R injury. Mechanistically, DUSP9 inhibits the phosphorylation of apoptosis signal-regulating kinase 1 (ASK1) by directly binding to ASK1, thereby decreasing tumor necrosis factor receptor-associated factor 6 (TRAF6), K63 ubiquitin and the phosphorylation of p38/JNK1 instead of ERK1. The present study documents a novel role of DUSP9 in hepatic I/R injury and implies the potential of targeting the DUSP9/ASK1 axis towards mitogen-activated protein kinase and TRAF6/inhibitor of κB kinase pathways.

## Introduction

Hepatic ischemia/reperfusion (I/R) injury comprises metabolic disorders and oxidative stress in the early stage, followed by late polarization, fibrosis, and apoptosis/necrosis [ 1, 2] . Recent studies have shown that different cell types might simultaneously regulate hepatic I/R injury in a synergistic or antagonistic manner. Blockade of CD8 ^+^ T cells leads to significant relief in hepatic I/R injury [ 3– 5] , while the function of CD4 ^+^ T cells seems more complicated [6]. Among the constituent cells in the liver, it appears that both parenchymal and nonparenchymal cells can guide stress signals, resulting in the progression of hepatic I/R injury [ 7, 8] . As liver-resident macrophages, Kupffer cells show high sensitivity to liver I/R injury and can mediate multiple stress signals contributing to the exacerbation of hepatic impairment according to previous studies [ 9, 10] . Therefore, targeting the transduction of stress signals in Kupffer cells is one of the directions for alleviating liver I/R injury.

Hyperactivated inflammation plays a key role as a connecting linkage in this continuous pathological process [11]. The inhibitor of the κB kinase (IKK) complex is one of the bridging proteins that regulates inflammatory signaling, as we previously demonstrated [ 12, 13] . IKK separates the inhibitory binding between NF-κB inhibitor α (IκBα) and nuclear factor kappa B (NF-κB) through phosphorylation (principally the catalytic subunit named IKKβ), thus releasing multiple cytokines and igniting the canonical IKK inflammatory signaling [14]. Therefore, we further investigated whether tumor necrosis factor (TNF) receptor-associated factor 6 (TRAF6)-induced K63 ubiquitination is the main upstream target of IKK in hepatic I/R injury.

Another inflammatory signaling pathway driving hepatic I/R injury is mitogen-activated protein kinase (MAPK), which is relatively independent of IKK [15]. Our previous studies indicated that the selective phosphorylation of MAPK subunits (including JNK, p38, and ERK) is determined by upstream diversity, either exogenous or endogenous; however, the proinflammatory and proapoptotic function of MAPK in the liver is not negligible [16]. Multiple classes of phosphorylation and ubiquitination in the MAPK cascade make the search for a suitable therapeutic target difficult. We previously demonstrated that F-box/WD repeat-containing protein 5 (Fbxw5) might be a central component of the E3 ligase upon apoptosis signal-regulating kinase 1 (ASK1), a pivotal kinase in MAPK [16]. However, more research is needed to identify molecules where IKK and MAPK intersect.

Dual specificity phosphatase 9 (DUSP9) is widely involved in innate immunity and can be regulated by ubiquitin [17]. DUSP9 protects the heart, lung, and breast by selectively repressing the phosphorylation of diverse MAPK or mechanistic targets of rapamycin kinase (mTOR) family members [ 18– 20] . Meanwhile, loss of DUSP9 leads to IKK complex excitation in nonalcoholic fatty liver disease (NAFLD), implying that a latent characteristic of DUSP9 crosses multiple inflammatory signals in the liver [21]. Prominently, our preliminary data delineated that DUSP9 might be negatively correlated with IKK in hepatic I/R injury. However, the exact function and mechanism of DUSP9 in hepatic I/R injury need to be analysed.

In this study, we aimed to explore the action of DUSP9 in excessive inflammation in hepatic I/R injury and further distinguish the internal mechanism. Our results suggested that DUSP9 diminished hepatic inflammation and apoptosis in the context of I/R. Alterations in protein expression showed that DUSP9 disrupted both the MAPK and IKK pathways. Notably, DUSP9 directly binds to ASK1, thus restraining ASK1 phosphorylation and K63 ubiquitination. The present study reveals how DUSP9 mitigates hepatic inflammation, thus providing novel insight into targets against hepatic I/R injury.

## Materials and Methods

### Study design

A standard mouse 70% warm hepatic I/R injury model was established according to our previous studies [ 22, 23] . Briefly, the conjunct blood vessel towards the left and middle lobes was clamped for 60 min of ischemia. The vascular clamp (M6333; MEYUE Bio, Shanghai, China) was then removed for reperfusion. The reperfusion time was set as 1, 3, 6, 9, or 12 h according to our experience [ 22, 23] . After comprehensive consideration of the relative expression levels of each group and previous research experience, we selected 1/6 h for I/R injury to carry out the follow-up experiment. Serum and tissue were collected after reperfusion. A classic hypoxia/reoxygenation (H/R) model was applied for cell simulation. Briefly, primary Kupffer cells were isolated using our previous improved extraction method [24], cultured in a tri-gas incubator for 6 h of hypoxia and then transferred to a conventional incubator for reoxygenation. The reoxygenation time was set as 3, 6, 12, or 24 h following our previous studies [ 25, 26] .

### Animal experiment

All animal experiment procedures were approved by the Animal Care and Use Committee of Chongqing Medical University. Male C57BL/6J mice aged 6–8 weeks (26.3–28 g bodyweight) were obtained from Chongqing Medical University Laboratory Animals Center, and housed under standard conditions of temperature, humidity, and day-night circulation. All mice had free access to food and water.

### Western blot analysis

Total protein was extracted using RIPA buffer (Beyotime, Shanghai, China) containing 10% protease inhibitor cocktail (Thermo Fisher Scientific, Waltham, USA), and the protein concentration was detected using a BCA kit (Beyotime, Shanghai, China). Then, protein samples were subject to 10% sodium dodecyl sulfate-polyacrylamide gel electrophoresis, and trantsferred to PVDF membranes. The membranes were blocked by QuickBlock (P0252, Beyotime) for 15 min, washed with TSBT for 3 times, and then incubated overnight at 4°C with the primary antibodies. After extensive wash, membranes were incubated with the corresponding horseradish peroxidase labeled goat anti-rabbit or goat anti-mouse IgG secondary antibodies (1: 2000; Beyotime). Fusion (SoloS EDGE, Influence, China) was used for visualization and grayscale calculation by chemiluminescence imaging. β-Actin was used as the loading control. The primary antibodies used are as follows: TRAF6 (1:5000; Abcam, Cambridge, UK), p-MKK7 (1:1500; Abcam), p-JNK1 (1:1500; Abcam), p-MAP kinase/ERK kinase 1/2 (MEK1/2) (1:2500; Abcam), interleukin (IL)-10 (1:1500; Abcam), IL-1β (1:1000; Abcam), c-Caspase-7 (1:500; Abcam), β-actin (1:2000; Beyotime, Shanghai, China), p-IKK (1:1000; Beyotime), IKK (1:1000; Beyotime), IKKβ (1:1500; Beyotime), p-IκBα (1:2500; Beyotime), IκBα (1:2500; Beyotime), p-p65 (1:2500; Beyotime), Bad (1:2000; Beyotime), p65 (1:1000; Beyotime), MKK7 (1:2000; Beyotime), TGFβ-activated kinase 1 (TAK1) (1:1000; Beyotime), Bcl2 (1:1000; Beyotime), p-TAK1 (1:1000; Beyotime), tumor necrosis factor α (TNF-α) (1:1000; Beyotime), MEK1/2 (1:3000; Beyotime), c-Caspase-3 (1:1000; Beyotime), DUSP9 (1:1000; Invitrogen, Carlsbad, USA), p-IKKβ (1:1000; Invitrogen), p-ERK1 (1:2000; Merck, Shanghai, China), JNK1 (1:1500; Zenbio, Chengdu, China), p-p38 (1:1000; Zenbio), p38 (1:500; Zenbio), ERK1 (1:1000; Zenbio), ASK1 (1:1000; Zenbio), p-ASK1(1:2000; Zenbio), p-MKK4 (1:1000; Zenbio), and MKK4 (1:1000; Zenbio).

### Hepatic pathological injury and apoptosis

Hepatic pathological injury and apoptosis were assessed based on our previous studies [ 12, 16] . Briefly, standard I/R liver injury comprising congestion, necrosis, and ballooning degeneration was evaluated by hematoxylin and eosin (HE) staining. Liver tissue was sampled and then fixed with paraformaldehyde for 24 h at 4°C. Apoptosis was evaluated using the terminal deoxynucleotidyl transferase-mediated deoxyguanosine triphosphate (dUTP) nick-end labelling (TUNEL) method. The tissue was stained using a Roche *in situ* Cell Death Detection Kit (Roche, Shanghai, China). Both HE and TUNEL images were observed with the Axio Imager A2m microscopic photographic system (Carl Zeiss, Oberkochen, Germany) and labelled using the ZEN2012 software (Blue edition; Carl Zeiss).

### Detection of cytokines and enzymes

Serum cytokines, including TNF-α, IL-1β, and IL-6, were measured using their corresponding enzyme-linked immunosorbent assay kits (Beyotime) as reported in our previous studies [ 27, 28] . According to the instructions, the kits were prepared in advance and left at room temperature for 15 min. After the reaction, 100 uL TMB chromogenic substrate was added to each well and incubated at 37°C in the dark for 15 min. Finally, stopping solution was added and the absorbance was immediately measured at 450 nm with a Multiskan Sky microplate reader (Thermo Fisher Scientific).

Serum alanine aminotransferase (ALT) and aspartate aminotransferase (AST) were monitored using a commercial microplate kit (JianCheng Bioengineering Institute, Nanjing, China) as reported in our previous studies [ 27, 28] . After the reaction, NaOH was added and incubated at room temperature for 10 min. Finally, the absorbance was measured at 510 nm or 505 nm with a Multiskan Sky microplate reader (Thermo Fisher Scientific).

### Quantitative real-time polymerase chain reaction (qRT-PCR)

The TRIzol reagent (Beyotime) was used for total RNA extraction as reported in our previous study [29]. Total RNAs were reverse transcribed into cDNA using SuperMix mRNA reverse transcription kit (Biomake, Shanghai, China). Then the relative mRNA levels of CXC chemokine ligand 10 (CXCL-10), C-C motif chemokine ligand 2, KH RNA binding domain containing, signal transduction associated 1 (Sam68), TNF-α, IL-1β, IL-10, arginase 1 (Arg-1), and CXCL-2 in the liver were measured by qRT-PCR (95°C for 5 min, including 1 cycle; 95°C for 15 s, 72°C for 20 s, and 40 cycles) using SYBR Green qPCR Master Mix (Biomake). mRNA levels were calculated using the 2 ^−ΔΔCt^ method and normalized to that of *β-actin*. Primers were designed and synthesized by Sangon (Shanghai, China) and the sequences are listed in Table 1.

**Table:** **Table 1** Sequences of primers used in this study

Gene	Primer sequence (5′→3′)
*IL-10*	Forward: AACCCAGGCACATCCGAAAAGC
	Reverse: AGAGACTACGCAGAGACCACAGAC
*IL-1β*	Forward: CCGTGGACCTTCCAGGATGA
	Reverse: GGGAACGTCACACACCAGCA
*Arg-1*	Forward: CTGCCTGCTTTCTGAGTGCTGAG
	Reverse: CCTGTGGTTCCGATAAGTGCTTCC
*CXCL-2*	Forward: ATGCCTGAAGACCCTGCCAAG
	Reverse: GGTCAGTTAGCCTTGCCTTTG
*MCP-1*	Forward: TTTTTGTCACCAAGCTCAAGAG
	Reverse: TTCTGATCTCATTTGGTTCCGA
*CXCL-10*	Forward: CAACTGCATCCATATCGATGAC
	Reverse: GATTCCGGATTCAGACATCTCT
*HRPT*	Forward: TCAACGGGGGACATAAAAGT
	Reverse: TGCATTGTTTTACCAGTGTCAA
*TNF-α*	Forward: GTCACCAGTTCCTCAGTTGTG
	Reverse: CACCTCCATTGTCCCTGTTTTAT
*Sam68*	Forward: GCCTACGGACAAGATGACTGGAATG
	Reverse: GATGCTCTCTGTATGCTCCCTTCAC
*β-Actin*	Forward: GGCTGTATTCCCCTCCATCG
	Reverse: CCAGTTGGTAACAATGCCATGT

### Transfection and drug administration *in vivo*

Adenovirus containing DUSP9 or short hairpin RNA (sh-RNA) of DUSP9 (sh-DUSP9; 5′-GCTCGAGATTCAGCCAATTTG-3′) or negative control (sh-NC; 5′-GTTCTCCGAACGTGTCACGT-3′) (GenePharma, Shanghai, China) was transfected *in vivo* via the tail vein(2.5×10 ^8^ pfu) 5 days before treatment. GS-4997, as a specific ASK1 inhibitor, was administered via oral gavage twice a day (15 mg/kg) 24 h and 12 h before treatment.

### Transfection and drug administration *in vitro*

Adenovirus was transfected into primary Kupffer cells *in vitro* 48 h before H/R treatment. The first medium exchange was conducted 6 h after transfection, and the second medium exchange was conducted 24 h after transfection. GS-4997 was administered 12 h before treatment at a concentration of 10 μM.

### Immunofluorescence analysis

Immunofluorescence analysis was performed as described in our previous studies [ 12, 13, 16] . Briefly, cells were uniformly inoculated on dishes. After H/R exposure, the supernatant was removed, and the cells were gently washed three times with PBS. After blocking and permeabilization, cells were incubated with primary antibodies overnight at 4°C. F4/80 was used as the marker of functional Kupffer cells. Primary antibody dilutions were set as follows: F4/80 (1:50; Abcam); p-p65 (1:150); p-IKK (1:200; Beyotime); TRAF6 (1:250; Beyotime). After extensive wash, cells were incubated with Cy3- or FITC-conjugated secondary antibodies (Beyotime) at 37°C for 45 min, followed by nuclear staining with DAPI (Beyotime). Immunofluorescence images was observed with a laser scanning confocal microscope (Olympus, Tokyo, Japan) and ZEN2012 software (Zeiss) was used for image acquisition and labelling.

### Immunoprecipitation analysis

Cells were lysed after treatment using a specific lysis buffer containing 25 mM HEPES, 150 mM NaCl, 1 mM EDTA, 2% glycerol, and 1 mM PMSF as previously reported [ 30, 31] . Samples were repeatedly oscillated and placed on ice for 30 min. After centrifugation at 12,000 *g* for 0.5 h at 4°C, the supernatant was collected, mixed with antibody-crosslinked protein G-agarose beads and incubated for 8 h at 4°C. Finally, proteins that bind to the beads were analysed by western blot analysis.

### Statistical analysis

Data are presented as the mean±SEM or the mean±SD. Experiments were performed at least in triplicates. The LSD t test was applied for result comparisons between 2 groups. One-way analysis of variance (ANOVA) followed by Bonferroni’s post hoc test was applied for result comparisons among multiple groups. *P* values of <0.05 were considered statistically significant. Calculations were performed using the Statistical Package for the Social Sciences (version 19.0; SPSS Inc, Chicago, USA).

## Results

### Overexpression of DUSP9 relieved hepatic I/R injury *in vivo*

**Figure 1:**
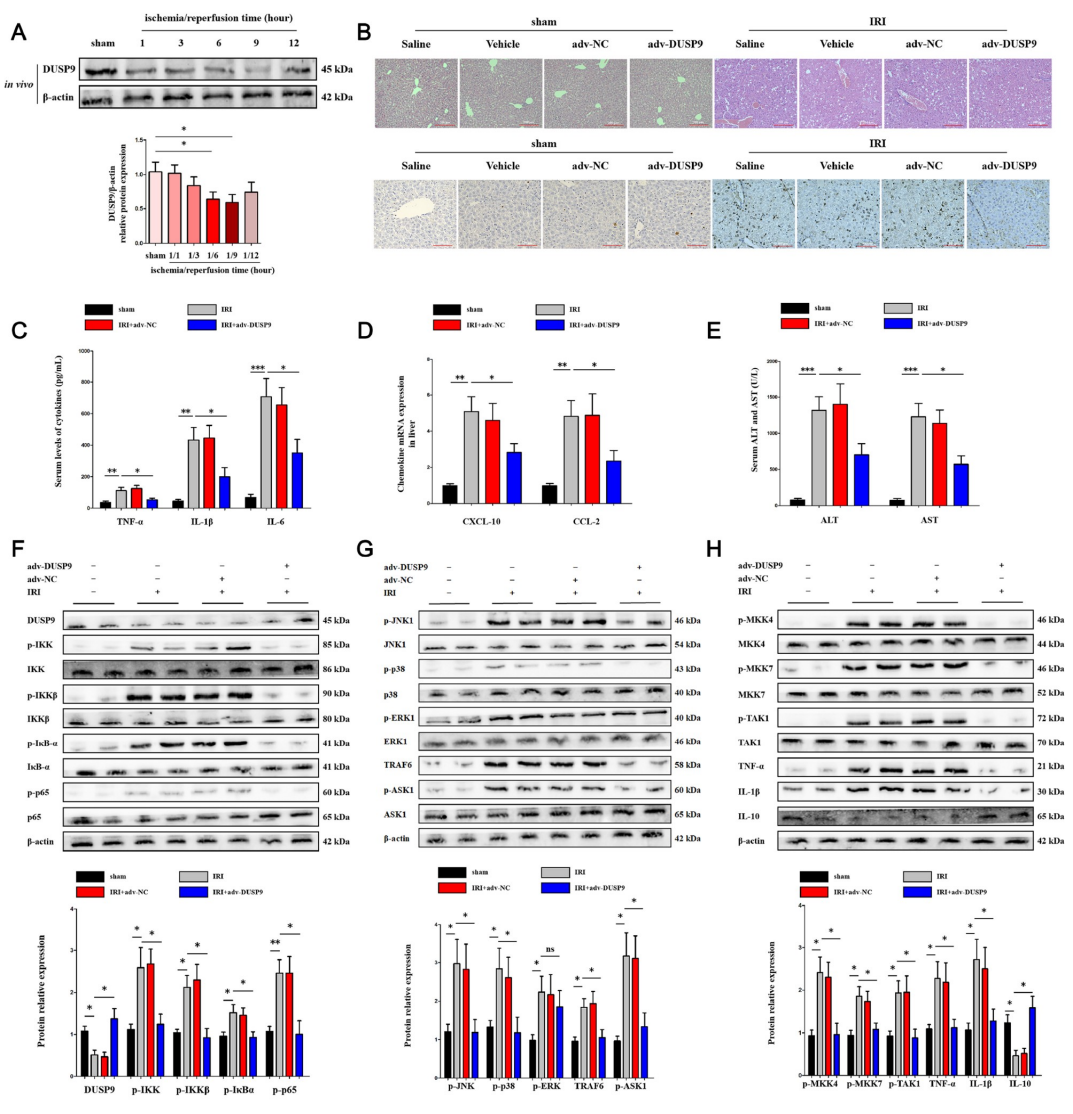
Figure 1 Enriched DUSP9 alleviated hepatic ischemia/reperfusion (I/R) injury (A) Protein expression of DUSP9 in primary Kupffer cells derived from I/R exposure in the liver. Ischemia for 1 h followed by reperfusion for 6 h was chosen for subsequent in vivo experiments. (B) The influence of DUSP9 overexpression on hepatic pathological changes (congestion, steatosis, and necrosis according to the Suzuki score, scale bar: 200 μm) and apoptosis (scale bar: 100 μm). (C–E) Release of cytokines, chemokines, and liver enzymes with increased DUSP9 in vivo. (F–H) Protein alterations of IKK, MAPK, and upstream/downstream molecules with increased DUSP9 in vivo. n=5–6 for each group. * P<0.05, ** P<0.01, *** P<0.001.

To explore the role of DUSP9, we first evaluated the expression of DUSP9 in hepatic I/R injury by western blot analysis. With prolonged reperfusion time, DUSP9 decreased gradually and reached the lowest level at 6 h and 9 h ( Figure 1A). Ischemia for 1 h followed by 6 h of reperfusion was then chosen for subsequent *in vivo* experiments. HE and TUNEL staining showed that DUSP9 overexpression resulted in less pathological injury and apoptosis, while little effect was observed in the physiological state ( Figure 1B). Similarly, the release of cytokines, chemokines, and liver enzymes was markedly suppressed with DUSP9 overexpression *in vivo* ( Figure 1C–E). Interestingly, western blot analysis results indicated that DUSP9 overexpression depleted both IKK/NF-κB and MKK/MAPK (JNK1, p38 but not ERK1) signaling pathways ( Figure 1F–H), which suggested that DUSP9 might restrain hepatic inflammation through limitation of these signaling pathways during hepatic I/R injury. In addition, overexpression of DUSP9 inhibited TRAF6, which is an important E3 ligand in liver I/R injury. These data indicated that DUSP9 might play an anti-inflammation role in hepatic I/R injury, while the underlying mechanism still needs further exploration.

### DUSP9 limited ASK1 phosphorylation-induced inflammation in Kupffer cells

**Figure 2:**
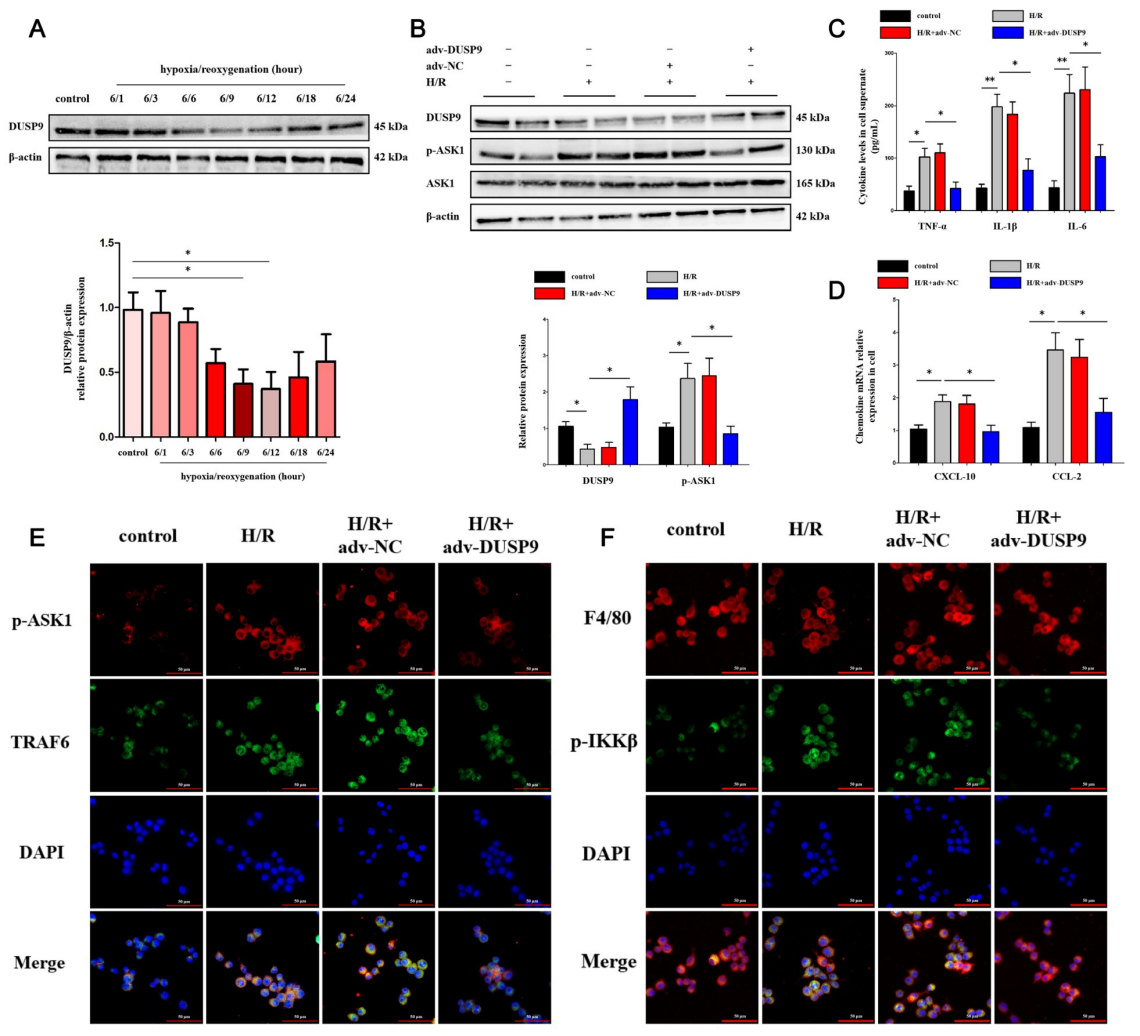
Figure 2 DUSP9 restrained ASK1 phosphorylation *in vitro* (A) Protein expression of DUSP9 in primary Kupffer cells treated with hypoxia/reoxygenation (H/R), a classical in vitro model of hepatic I/R injury. Hypoxia for 6 h followed by reoxygenation for 12 h was chosen for subsequent in vitro experiments. (B) Influence of DUSP9 accumulation on ASK1 phosphorylation. (C,D) Release of cytokines and chemokines in vitro. (E,F) Nuclear translocation of NF-κB p65 and relative intensity of TRAF6 in primary Kupffer cells detected by immunofluorescence analysis. Scale bar: 50 μm. * P<0.05, ** P<0.01.

To further explore how DUSP9 functions, we investigated whether DUSP9 exhibits similar anti-inflammatory characteristics *in vitro*. A classic hypoxia/reoxygenation (H/R) model was applied for *in vitro* stimulation. DUSP9 expression gradually decreased and reached the lowest level at 9 h and 12 h with extended reoxygenation time ( Figure 2A). Consistent with the *in vivo* results, DUSP9 accumulation *in vitro* reduced ASK1 phosphorylation in primary Kupffer cells exposed to H/R ( Figure 2B). We then analysed the levels of cytokines and chemokines to determine inflammatory progression. Likewise, the release of cytokines ( Figure 2C) and chemokines ( Figure 2D) was significantly decreased in the DSUP9 overexpression groups. Immunofluorescence analysis showed an intuitively impaired intensity of TRAF6 and IKKβ with DUSP9 overexpression ( Figure 2E,F). These data suggested that DUSP9 might limit inflammation by inhibiting ASK1 phosphorylation.

### DUSP9 overexpression restrained MAPK and IKK pathway proteins in Kupffer cells

**Figure 3:**
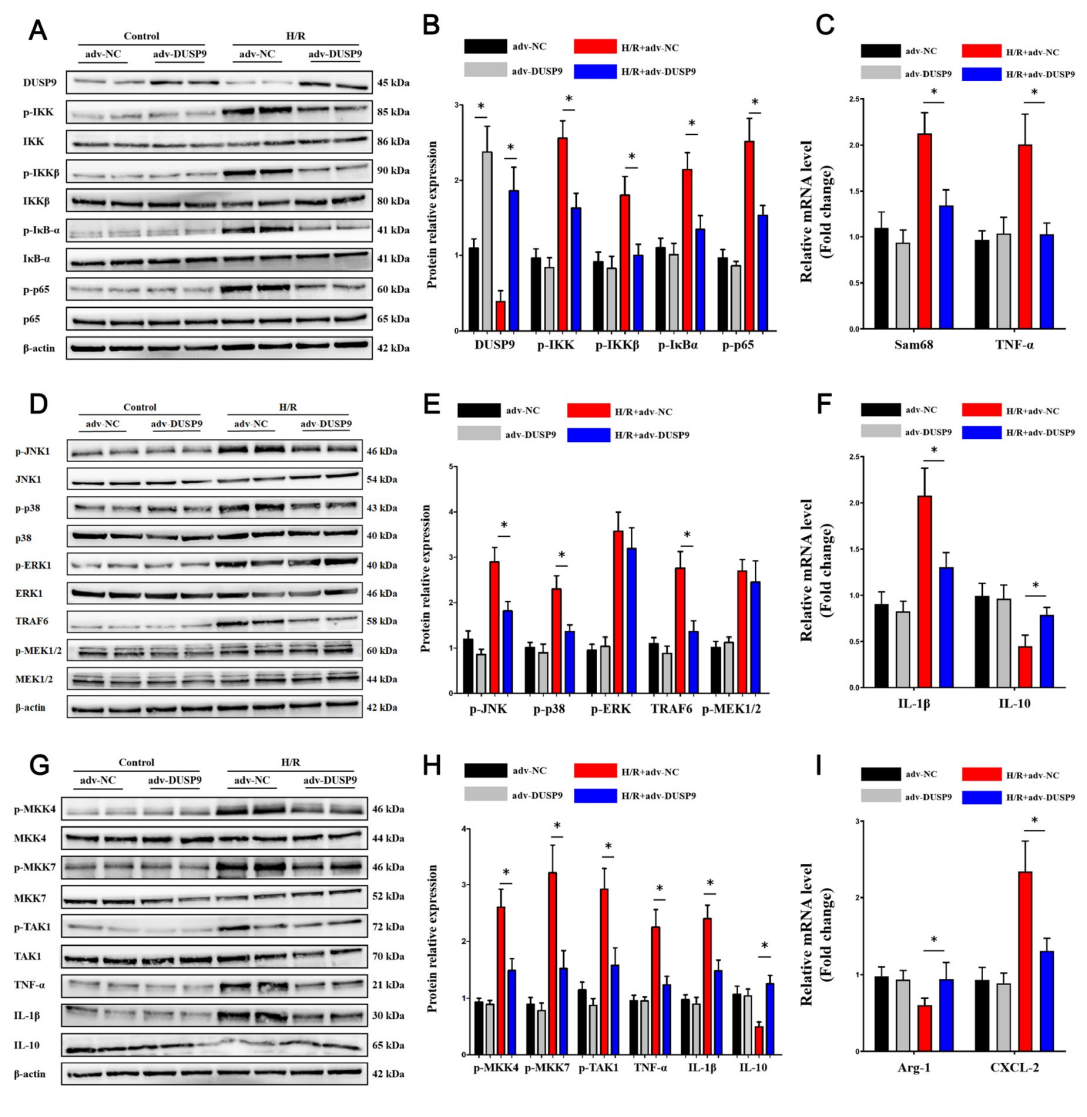
Figure 3 Reinforcement of DUSP9 *in vitro* suppressed MAPK and IKK signaling pathway activation (A–C) DUSP9 inhibited the canonical IKK complex-guided NF-κB pathway in hepatic I/R injury but had little effectiveness in a physiological state. (D–F) DUSP9 repressed JNK1 and p38 phosphorylation but not ERK1 phosphorylation in hepatic I/R injury. (G–I) Protein changes in MKKs and cytokines in hepatic I/R injury or the physical state upon DUSP9 enrichment. * P<0.05.

To further verify the role of DUSP9 *in vitro*, the effects of DUSP9 on the IKK and MAPK axes were evaluated. Overexpression of DUSP9 decreased the protein expression of the IKK complex-guided canonical NF-κB signaling pathway ( Figure 3A–C). Notably, DUSP9 impaired JNK1 and p38 phosphorylation, but not ERK1 phosphorylation, among the MAPK subunits ( Figure 3D–F). Although ERK1 activation was elevated in the model, alteration of DUSP9 did not significantly affect ERK1, suggesting that ERK1 might be relatively independent of DUSP9 in liver I/R injury. We further tested critical upstream and downstream proteins. As expected, the activation of MKK and TAK1 was decreased by DUSP9 overexpression *in vitro* ( Figure 3G–I). Consistent with the above findings, TRAF6 expression ( Figure 3D) and K63 ubiquitination ( Supplementary Figure S1B) were also inhibited by DUSP9 *in vitro*, indicating a negative regulatory role of DUSP9 against TRAF6-guided ubiquitination.

### *DUSP9* knockdown facilitated both MAPK and IKK signaling pathway proteins in Kupffer cells

**Figure 4:**
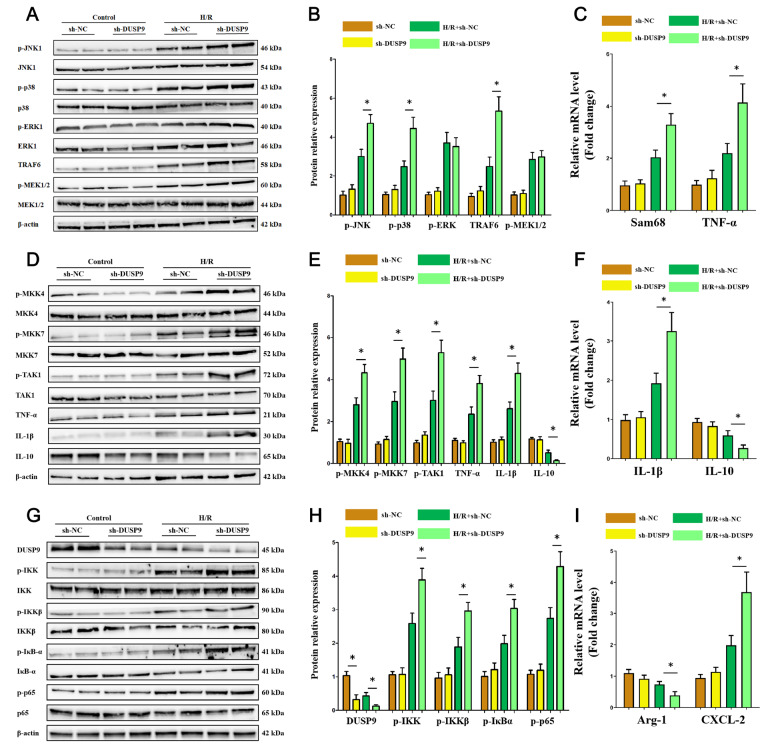
Figure 4 DUSP9 knockdown enhanced MAPK and IKK activation *in vitro* (A–C) DUSP9 deficiency led to JNK1 and p38 but not ERK1 activation. DUSP9 has little effect on MAPK pathway proteins in the physiological state. (D–F) DUSP9 reduction in vitro increased the protein levels of MKKs and cytokines. (G–I) DUSP9 deficiency promoted IKK pathway proteins and IκBα/NF-κB under hepatic I/R injury. * P<0.05.

Given the protective function of DUSP9 as previously mentioned, we downregulated DUSP9 expression *in vitro* for further verification. As shown in Figure 4A–C, DUSP9 deficiency selectively facilitated MAPK signaling pathway proteins. Phosphorylation of JNK1 and p38 was increased when *DUSP9* was knocked down in H/R-treated Kupffer cells. Consistent results were obtained among related upstream and downstream proteins ( Figure 4D–F). Similarly, although p-ERK1 was elevated in the model, changes in DUSP9 did not affect its protein activation afterwards. Meanwhile, a more intense hyperactivation of canonical IKK/NF-κB was detected in the *DUSP9* knockdown groups ( Figure 4G–I). These data indicated that loss of DUSP9 facilitates both MAPK and IKK signaling pathways simultaneously.

### DUSP9 attenuated ASK1 phosphorylation by directly binding to ASK1

**Figure 5:**
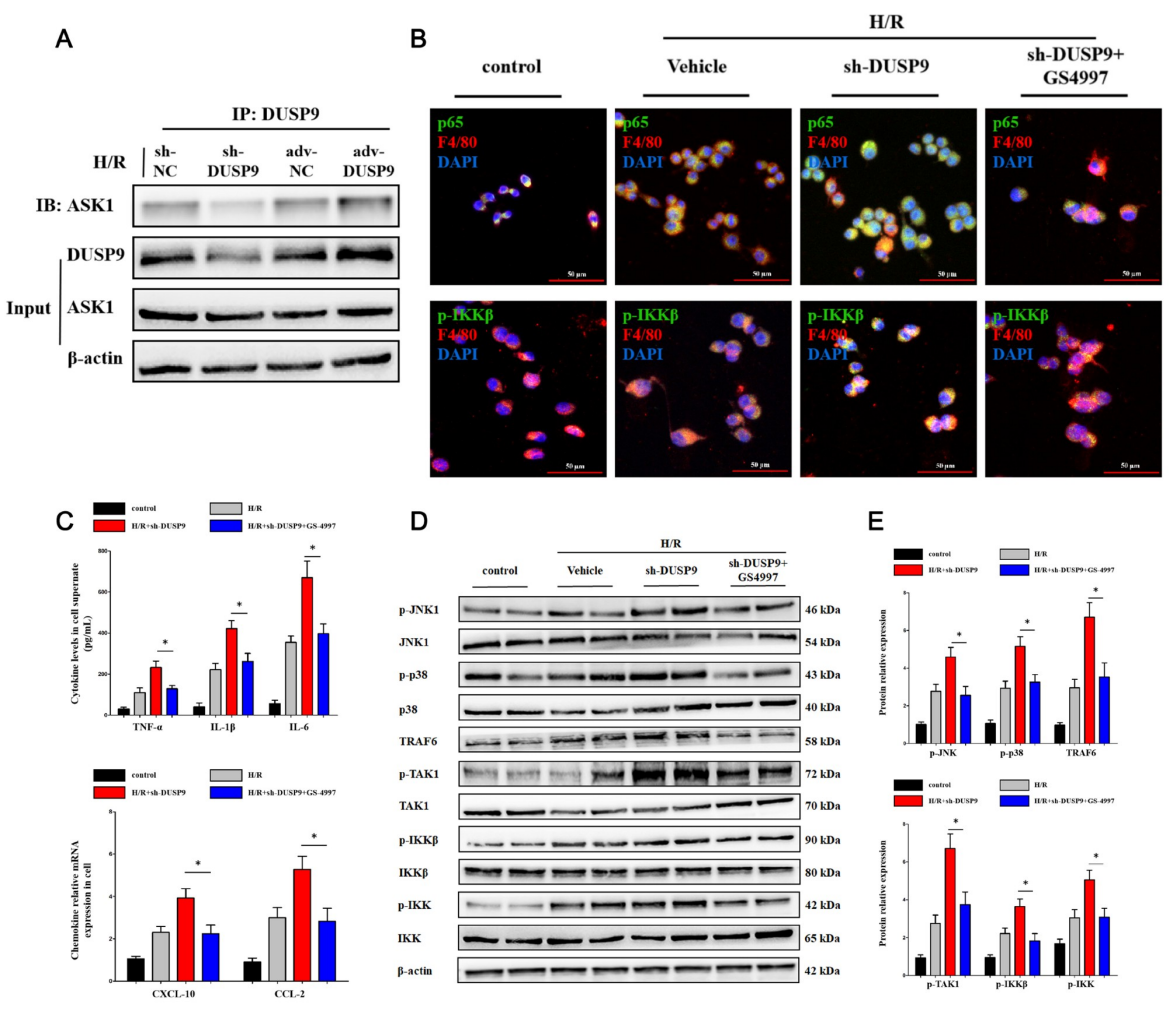
Figure 5 DUSP9 directly interacted with ASK1 (A) The binding strength of DUSP9 to ASK1 was enhanced with a reoxygenation time extension. (B) DUSP9 inhibition restricted NF-κB nuclear translocation and TRAF6 relative fluorescence intensity but was abolished by a specific ASK1 inhibitor (GS-4997). (C) The release of cytokines and chemokines in vitro in the presence of GS-4997. (D,E) Inhibition of ASK1 abrogated MAPK and IKK phosphorylation derived from DUSP9 deficiency. * P<0.05.

To explore the underlying mechanism of DUSP9, the molecular connection between DUSP9 and ASK1 was further investigated by immunoprecipitation analysis. Impressively, DUSP9 was found to directly interact with ASK1, thus inhibiting K63 ubiquitination and ASK1 phosphorylation ( Figure 5A and Supplementary Figure S1A,B). This implied that DUSP9 restricted inflammation in hepatic I/R injury through direct binding to ASK1, thereby reducing ASK1 activation. We then wondered whether ASK1 is the major downstream target of DUSP9 in hepatic I/R injury. A specific ASK1 inhibitor, named GS-4997, was utilized *in vitro* according to our previous experience. Importantly, GS-4997 was able to abolish p65 translocation and IKK activation derived from *DUSP9* knockdown ( Figure 5B). The release of cytokines and chemokines was decreased GS-4997 treatment ( Figure 5C). A significant reduction in MAPK and IKK protein was confirmed in the ASK1 inhibition group based on *DUSP9* knockdown ( Figure 5D,E), implying that DUSP9 alleviates inflammation in an ASK1-dependent manner. These data manifested that DUSP9 might function in hepatic I/R injury through a direct interaction with ASK1.

### Inhibition of ASK1 abrogated the proinflammatory consequence of DUSP9 deficiency *in vivo*

**Figure 6:**
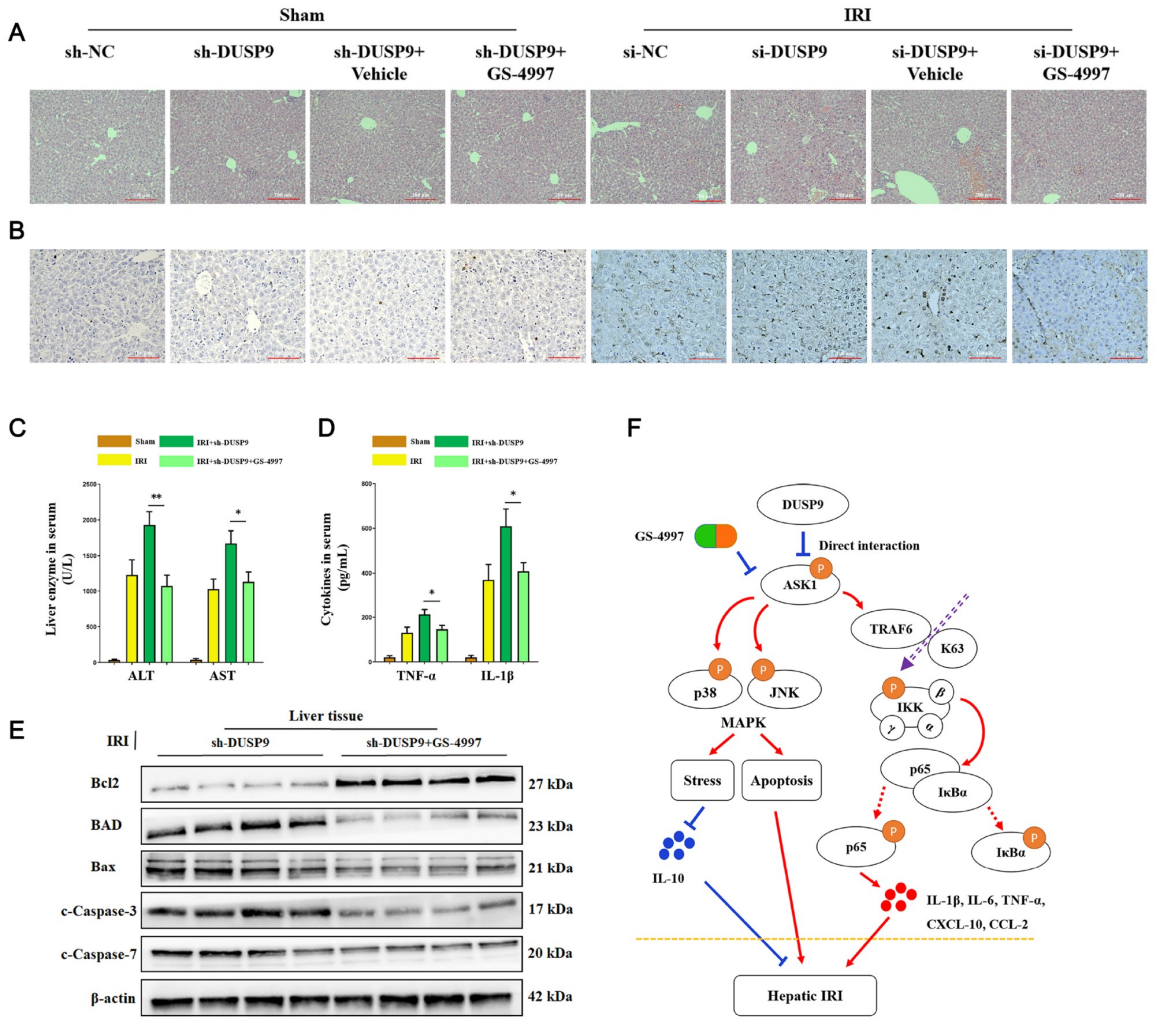
Figure 6 ASK1 inhibition abolished the proinflammatory consequences derived from DUSP9 deficiency *in vivo*. (A) GS-4997 suppressed pathological lesions, including congestion, steatosis, and necrosis in the liver. Scale bar: 200 μm. (B) ASK1 repression mitigated hepatic apoptosis in hepatic I/R injury. Scale bar: 100 μm. (C,D) Release of liver enzymes and cytokines was detected by the microplate method. (E) IKKβ activation in primary Kupffer cells induced by I/R exposure and GS-4997 treatment. Scale bar: 50 μm. (F) Molecular mechanism by which DUSP9 alleviates hepatic I/R injury. n=6 for each group. * P<0.05, ** P<0.01.

To further evaluate the potential efficacy of pharmacological interventions, the function of GS-4997 in resisting DUSP9 deficiency *in vivo* was further investigated. Pathological changes and apoptosis in the liver were relieved by GS-4997 treatment ( Figure 6A,B). Moreover, ASK1 inhibition obviously diminished the release of liver enzymes and cytokines ( Figure 6C,D). Immunofluorescence analysis showed weaker TRAF6 activation when ASK1 was restricted under DUSP9 deficiency ( Figure 6E). Collectively, we concluded that DUSP9 decreased both the MAPK and IKK pathways by interacting with ASK1, thereby restraining ASK1 phosphorylation and TRAF6 in hepatic I/R injury ( Figure 6F). These data indicated that pharmacologic interventions towards ASK1 might be effective, which could be an alternative strategy for future clinical translation.

## Discussion

Effective treatment for hepatic I/R injury is still lacking due to inadequate suitable therapeutic targets [ 31– 33] . In the present study, we revealed the protective role of DUSP9 in the liver both *in vivo* and *in vitro*. Hepatic I/R injury depleted endogenous DUSP9 over a prolonged period, accompanied by IKK and MAPK activation. Similar pathway activation has been observed in our and other studies [ 12, 13, 34] , both of which are supposed to be critically and relatively independent in hepatic I/R injury [ 15, 16] .

The IKK complex consists of two catalytic subunits (IKKα and IKKβ) and one regulatory subunit (IKKγ) [35]. A previous study reported that phosphorylation of IKKβ was principally involved in hepatic I/R injury instead of IKKα or IKKγ [34], which was consistent with our previous reports [ 12, 13] . A specific terminal domain (NEMO binding domain, NBD) shared by IKKα or IKKβ could directly bind to IKKγ, thereby phosphorylating downstream IκBα. Importantly, our unpublished data identified the noncanonical IKK pathway induced by cytoplasmic transfer of IKKγ, which might participate in maintaining the IKK complex in response to I/R injury. The present study showed that DUSP9 could suppress TRAF6 expression and K63 ubiquitination, thus inhibiting IKKβ instead of IKKα. We have previously revealed the activation selectivity of the two catalytic subunits of IKK; however, we failed to identify the key molecule that specifically regulates IKKa [ 12, 16] . Moreover, we did not perfrom TRAF6 interference to further demonstrate its specific role in the regulation of DUSP9 in liver I/R injury, which is an area we will explore in the future.

Subunit selectivity has also been observed in the MAPK superfamily. JNK and p38 are the dominant participants that exacerbate hepatic I/R injury [36], whereas ERK is more often involved in hepatic development and terminal fibrosis but rarely in the intermediate processes [37]. According to our previous results, the diverse activation of MAPK subunits depends on disparate upstream molecules and functional cell types [ 13, 16] . Nonparenchymal cells, such as Kupffer cells, are signal receiving and amplifying devices in hepatic I/R injury that recognize inflammatory signals, including JNK and p38 phosphorylation, thereby making parenchymal cells take effect [ 38, 39] . Herein, ASK1 was found to be the direct target of DUSP9 in hepatic I/R injury, which led to the inhibition of ASK1 and MKKs, thereby ultimately remising JNK1 and p38. Although this interaction might contribute to DUSP9-guided IKK and MAPK suppression, the major enzymes that are involved have not been specifically demonstrated. Meanwhile, the IKK family itself consists of two catalytic subunits and one regulatory subunit. Whether DUSP9 affects IKKβ directly or indirectly through TRAF6 remains to be further investigated. Our functional results are consistent with those concentrating on ASK1 in other inflammatory models, while DUSP9-ASK1 complex authentication in hepatic I/R injury is relatively innovative [ 40, 41] . However, we failed to use other specific inhibitors of MAPK or IKK subunits to further explore the mechanisms after pathway rescue, which is a limitation of this study. We will further explore the specific mechanisms by which DUSP9 regulates these two pathways in the future. Another limitation of the present study is the lack of real-world clinical analysis. As a regulator of TRAF6/IKK in addition to MAPK [42], the efficacy of ASK1 drugs against NAFLD is still unclear in some clinical studies [ 43, 44] . The results of clinical studies on ASK1 inhibitors in NAFLD are gradually emerging, but their clinical application in liver I/R injury is relatively inadequate with regard to their target diversity. Some public databases and sequencing data could better reveal the characteristics of ASK1 in hepatic I/R injury.

Kupffer cells are the largest pool of nonparenchymal cells in the liver and contribute to the inflammatory progression of liver I/R injury [ 45, 46] . Although parenchymal cells in the liver are far ahead in number, Kupffer cells could partially determine the outcome of liver I/R injury due to their unique anatomical distribution and physiological functions [ 38, 47] . In this study, we found that the DUSP9-mediated inflammatory response in Kupffer cells might ultimately result in the initiation of hepatic apoptosis signaling, but we did not further explore the influence of DUSP9 alterations in hepatocytes, which could be a future direction. At the same time, the use of other macrophage cell lines (such as the RAW 264.7-cell line) *in vitro* might further strengthen our conclusion.

In conclusion, we identified that DUSP9 is a latent modulator that alleviates hepatic I/R injury by inhibiting ASK1 phosphorylation. DUSP9 enrichment decreases both TRAF6/IKK and MAPK inflammatory pathway proteins. Our results delineated a potential therapeutic axis by which DUSP9/ASK1 controls the inflammatory response in hepatic I/R injury.

## Supporting information

083Table1

083Table2

## Supplementary Data

Supplementary data is available at *Acta Biochimica et Biophysica Sinica* online.
