# Supplementary material for: DUSP9 alleviates hepatic ischemia/reperfusion injury by restraining both mitogen-activated protein kinase and IKK in an apoptosis signal-regulating kinase 1-dependent manner: Role of DUSP9 in hepatic ischemia/reperfusion injury
Source: Acta Biochim Biophys Sin (Shanghai). 2022 Dec 22;54(12):1811–21. doi: 10.3724/abbs.2022183 (PMC10157530; doi:10.3724/abbs.2022183)
Supplement: 083Table1 [file 083Table1.pdf]

Table 1 Antibody dilutions for western blot analysis

Antibody name	Dilution
TRAF6	1/ 5000
MEK1/2	1/ 3000
p-p65, p-IκBα, IκBα, p-MEK1/2	1/ 2500
β-actin, MKK7, p-ASK1, p-ERK1, Bad, Bax	1/ 2000
p-MKK7, JNK1, IKKβ, IL-10	1/ 1500
DUSP9, p65, p-IKKβ, IKK, p-IKK, IL-1β, p-TAK1, TAK1, ASK1, p-p38, ERK1, Bcl2, TNF-α, c-Caspase3	1/ 1000
p-JNK1, p38	1/ 500

TRAF6, p-MKK7, p-JNK1, p-MEK1/2, IL-10, IL-1β, c-Caspase-7 were from Abcam, Cambridge, UK; β-actin, p-IKK, IKK, IKKβ, p-IκBα, IκBα, p-p65, Bad, p65, MKK7, TAK1, Bcl2, p-TAK1, TNF-α, MEK1/2, c-Caspase-3 were from Beyotime, Shanghai, China; DUSP9, p-IKKβ (Invitrogen, Carlsbad, USA), p-ERK1 were from Merck, Shanghai, China; and JNK1, p-p38, p38, ERK1, ASK1, p-ASK1, p-MKK4, and MKK4 were from Zenbio, Chengdu, China.
