# Supplementary material for: DUSP9 alleviates hepatic ischemia/reperfusion injury by restraining both mitogen-activated protein kinase and IKK in an apoptosis signal-regulating kinase 1-dependent manner: Role of DUSP9 in hepatic ischemia/reperfusion injury
Source: Acta Biochim Biophys Sin (Shanghai). 2022 Dec 22;54(12):1811–21. doi: 10.3724/abbs.2022183 (PMC10157530; doi:10.3724/abbs.2022183)
Supplement: 083Table2 [file 083Table2.pdf]

**Table 2. Sequences of primers used in this study**

| Gene | Primer sequence (5′→3′) | |
|---|---|---|
| *IL-10* | Forward | AACCCAGGCACATCCGAAAAGC |
| | Reverse | AGAGACTACGCAGAGACCACAGAC |
| *IL-1β* | Forward | CCGTGGACCTTCCAGGATGA |
| | Reverse | GGGAACGTCACACACCAGCA |
| *Arg-1* | Forward | CTGCCTGCTTTCTGAGTGCTGAG |
| | Reverse | CCTGTGGTTCCGATAAGTGCTTCC |
| *CXCL-2* | Forward | ATGCCTGAAGACCCTGCCAAG |
| | Reverse | GGTCAGTTAGCCTTGCCTTTG |
| *MCP-1* | Forward | TTTTTGTCACCAAGCTCAAGAG |
| | Reverse | TTCTGATCTCATTTGGTTCCGA |
| *CXCL-10* | Forward | CAACTGCATCCATATCGATGAC |
| | Reverse | GATTCCGGATTCAGACATCTCT |
| *HRPT* | Forward | TCAACGGGGGACATAAAAGT |
| | Reverse | TGCATTGTTTTACCAGTGTCAA |
| *TNF-α* | Forward | GTCACCAGTTCCTCAGTTGTG |
| | Reverse | CACCTCCATTGTCCCTGTTTTAT |
| *Sam68* | Forward | GCCTACGGACAAGATGACTGGAATG |
| | Reverse | GATGCTCTCTGTATGCTCCCTTCAC |
| *β-Actin* | Forward | GGCTGTATTCCCCTCCATCG |
| | Reverse | CCAGTTGGTAACAATGCCATGT |
